# Treatment of the Enlarged Clitoris

**DOI:** 10.3389/fped.2017.00125

**Published:** 2017-08-28

**Authors:** Martin Kaefer, Richard C. Rink

**Affiliations:** ^1^Pediatric Urology, Indiana University School of Medicine, Indianapolis, IN, United States

**Keywords:** congenital adrenal hyperplasia, clitoromegally, clitoroplasty, disorders of sex development, history

## Abstract

Management of the enlarged clitoris, because of its import for sexual function, has been and remains one of the most controversial topics in pediatric urology. Early controversy surrounding clitoroplasty resulted from many factors including an incomplete understanding of clitoral anatomy and incorrect assumptions of the role of the clitoris in sexual function. With a better understanding of anatomy and function, procedures have evolved to preserve clitoral tissue, especially with respect to the neurovascular bundles. These changes have been made in an effort to preserve clitoral sensation and preserve orgasmic potential. It is the goal of this manuscript to describe the different procedures that have been developed for the surgical management of clitoromegally, with emphasis on the risks and benefits of each. Equally important to any discussion of such a sensitive topic is an understanding of long-term patient outcomes. As we will see, despite its importance, there has been a dearth of data in this regard. Future work in the arena of patient satisfaction will undoubtedly play a major role in directing our surgical approach.

## Anatomy

The clitoris, like the penis, consists of two corpora cavernosa. While not having a defined corpora spongiosum, the clitoris does have the male equivalent of the glans, which consists of spongiosal tissue. Arterial supply branches from the internal pudendal artery that travels *via* Alcock’s canal near the ischial tuberosity. These arteries course ventrally and are on the medial aspect of the bifurcated corpora, where they then course dorsally along the phallic shaft (1). Innervation governing tumescence and sensation are also similar to that observed in the penis. Most important to the discussion of clitoral surgery is the anatomic course of the nerves that provide sensation to the glans. The clitoral neurovascular bundles ascend along the ischiopubic rami and meet as paired bundles that course along the dorsal surface and then pass largely intact into the glans. There is a notable absence of nerves at both the 12 o’clock position and the ventral aspect of the shaft (2, 3).

In considering the anatomy of the clitoris, one should also be versed in the anatomy of neighboring structures. If clitoroplasty surgery is undertaken, it is done so with the intent of providing an appearance that aims to closely resemble the typical female phenotype. Regression of the glans clitoris, with creation of a labia minora and clitoral hood to properly conceal the glans, requires a good understanding of these later structures. A recent study of non-CAH females revealed that the labia minora converge under the clitoral glans, separate to the clitoral hood. Variability exists with regard to the morphology of the clitoral hood. The four recognized morphologies include horseshoe, trumpet, coffee bean, and tent (4).

## Etiology

There are a limited number of clinical entities that result in clitoromegally. The most common condition to cause enlargement of the clitoris is the 46XX DSD condition congenital adrenal hyperplasia (CAH). A number of enzymatic defects in the production of cortisol can cause a shunting of cortisol precursors to an alternate metabolic pathway, which results in an excess production of adrenal androgens (i.e., DHEA, androstenedione, and testosterone). Females with classic CAH, whether salt-losing or non-salt-losing, present at birth with an enlarged phallus due to *in utero* exposure to excess fetal adrenal androgens. The overall worldwide incidence of classic CAH is one in 15,000 live births of which two-thirds are salt wasting (5).

In 1950, Lawson Wilkins demonstrated that it was possible to suppress adrenal androgen production by providing these individuals with cortisone. With the ability to abrogate ongoing hormonal stimulation of the phallus, it was no longer deemed necessary to reconstruct these children as males. The evolution of surgery to “feminize” the appearance of the genitalia thus began.

A far less common condition that can cause enlargement of the phallus in the genetic female is exogenous *in utero* androgen exposure. This can either be due to the pregnant mother suffering from masculinizing tumors such as arrhenoblastoma [Ovarian Sertoli-Leydig cell tumors (SLCTs)] of the ovary or exogenous intake of androgenic hormones (6–8).

A number of Disorders of Sex Development (DSD) possessing a cell line with a Y chromosome can result in a child with an enlarged phallus who may be assigned the female gender role. This choice should only be undertaken after extensive multidisciplinary assessment and counseling of the parents. In Sex Chromosome Mosaicism DSD (previously labeled mixed gonadal dysgenesis), the karyotype 45X/46XY can lead to a child having an epididymis, vas deferens, and testicle on one side of the body while having a hemivagina, hemiuterus, fallopian tube, and non-functional streak ovary on the contralateral side (9, 10). In ovotesticular DSD (previously labeled true hermaphroditism), functional ovarian and testicular tissues are both identifiable. In both conditions, the phallus will undergo some degree of enlargement due to endogenous testosterone production. While it is beyond the scope of this manuscript to describe the process of assigning gender to these individuals, if a female gender role is selected then surgery to render the phallus more clitoral in appearance may be entertained (11).

Rare cases of females with clitoromegally secondary to pelvic plexiform neurofibroma have also been reported (12). Finally, it is important to recognize that idiopathic clitoromegally can present in females that are born extremely prematurely (<28 weeks gestational age) (13). This is felt most likely due to fetal programming causing a surge in LH and the overactivation of the pituitary–gonadal axis (14).

## Surgical Procedures

### General

At present, the decision to perform genital surgery in children with clitoromegally is intensely debated. As with all reconstructive surgery for patients with Disorders of Sex Development (DSD), three specific reasons for intervening are typically considered: providing anatomy suitable for penile–vaginal intercourse, achieving a manner for urination commensurate with gender identity (i.e., sitting for females, standing for males), and providing a phenotypical appearance that resembles the assigned gender (11). Since the only known function of the clitoris itself is to provide sexual pleasure, the later goal is the only one that is relevant to the discussion of clitoroplasty.

The primary concern in performing surgeries that address clitoral enlargement is that the procedure may reduce innervation to the clitoris. To this point, it is important to note that when compared to controls, sexually mature females who have undergone surgery in childhood frequently report reduced sensation and decreased ability to achieve orgasm (15–17). As a result of these potential risks, the Chicago consensus and the Endocrine Society guidelines on the management of CAH have recommended that clitoral surgery should be postponed in girls with mild degrees of clitoromegaly (<2 cm) (18, 19). In cases of moderate and severe virilization, both guidelines recommended that clitoroplasty be considered as long as an experienced surgeon performs it.

Most clitoral surgery is performed in conjunction with a vaginoplasty. Feminizing genitoplasty (clitoroplasty + vaginoplasty) is often undertaken in the first year of life for children with low and midlevel confluences of the urogenital sinus. The advantage of performing both procedures simultaneously is that the common urogenital sinus can be used for a host of reconstructive purposes (20). If vaginoplasty is not undertaken simultaneously, one should always use the sinus tissue to create a more female-like vulva.

### Perioperative Considerations

#### Endocrine Management

Prior to embarking on major surgery such as clitoroplasty, the patient must be in optimal physiologic condition. In that the majority of patients who present with clitoromegally are patients with CAH, an understanding of proper endocrine management in these patients is imperative.

Congenital adrenal hyperplasia due to 21-hydroxylase deficiency accounts for 95% of cases and shows a wide range of clinical severity depending on the degree of impairment of cortisol and aldosterone biosynthesis (21). As a general rule, there is high concordance between genotypic CYP21A2 mutations and phenotype (22). In the most severe form, concomitant aldosterone deficiency leads to salt loss. The three clinical phenotypes are typically classified as classic salt-losing (most severe), classic non-salt-losing (simple-virilizing), or non-classic (mild or late-onset). In childhood, treatment is geared toward optimizing growth and pubertal development. Once adult height is achieved, treatment should be focused on optimizing fertility and quality of life and minimizing the side effects of glucocorticoid therapy.

Treatment of the classic or severe form of CAH requires suppression of adrenal androgen overproduction and replacement of cortisol and aldosterone. The medical treatment of CAH is challenging and necessitates “walking a fine line” between glucocorticoid excess and hyperandrogenism.

Non-classic CAH is a milder form of the disease. Although the same gene, CYP21A2, is involved in both the severe and mild forms, genetic mutations typically associated with non-classic CAH result in substantially less impairment of 21-hydroxylase activity. Thus, patients with non-classic CAH do not have cortisol deficiency but instead may have manifestations of hyperandrogenism, later in childhood or in early adulthood. Treatment of the mild or non-classic form is targeted at controlling excess androgen symptoms and may or may not involve glucocorticoid therapy.

The Pediatric Endocrine Society and the European Society for Pediatric Endocrinology recommend glucocorticoid dosing for children in the form of hydrocortisone 10–15 mg/m^2^/day divided three times daily (23). Longer-acting glucocorticoids have typically been avoided in children due to their potential for growth suppression (24). No consensus exists for glucocorticoid dosing in adults. Clinicians may use hydrocortisone, prednisone, prednisolone, dexamethasone, or a combination of treatments. Long-acting glucocorticoids are preferable because they are effective given once or twice daily (25). The specific regiment varies between institutions worldwide. In a survey in the United Kingdom of 30 teaching centers, a variety of different regimens were utilized; hydrocortisone was the most common, followed by dexamethasone and then prednisolone (26).

Mineralocorticoid is given in classic cases of CAH. Fludrocortisone is provided to maintain normal electrolyte and plasma renin activity. The use of fludrocortisone is also recommended in simple-virilizing CAH and allows management with lower doses of glucocorticoid (23). Some clinicians feel that the same daily dose of fludrocortisone given in split doses twice daily is more effective than once daily therapy (25). Overtreatment should be avoided and may result in hypertension.

Levels of 17-hydroxyprogesterone, testosterone, androstenedione, and plasma renin activity are used to evaluate adequacy of therapy and patient compliance. The suggested target 17-OHP range for children with CAH is 400–1,200 ng/dl (21). Adrenal androgen concentrations later in the day and after medication will be lower, and target levels for hormones measured in this manner are unknown; thus, hormones are best measured early in the morning and before medication. The target goal for androstenedione, testosterone, and plasma renin values are to have them within the normal range for age. Bone age and somatic growth data are also used to determine the efficacy of the chosen steroid replacement regimen.

#### Stress Steroid Dosing

The physiologic stress that surgery induces requires thoughtful increases in perioperative steroid dosing. Preoperative dosing consists of a single dose of hydrocortisone 100 mg/m^2^ IV immediately before surgery followed by this same dose given IV divided q6 hours for at least 24 h.

#### Perioperative Counseling

The role that clitoral surgery plays in clitoromegally remains strongly debated. In addition, the emotional impact of having a child with clitoromegally will be extremely challenging for most families. It is for this reason that it is imperative that a multidisciplinary care model be utilized to provide comprehensive care of the child and family. The 2002 consensus statement by the Lawson Wilkins Pediatric Endocrine Society and the European Society of Pediatric Endocrinology recommends that “A well-organized multidisciplinary team (including specialists in pediatric endocrinology, psychosocial services, pediatric surgery/urology, and genetics) is essential…” and “it is important that the coordinator of the team has an experience in the long-term care of the patient with CAH and provides a consistent message to patients” (23). Specific evidenced-based models for multidisciplinary care have been published (27). Evidence supports that laboratory monitoring for appropriate steroid dosing and the provision of mental health care occurs more consistently when these multidisciplinary models are in place.

### Specific Procedures

Surgical techniques for management of clitoromegaly can broadly be divided into three categories: clitorectomy, reduction clitoroplasty, and corporal-sparing techniques.

#### Clitorectomy

Prior to 1970, many prominent surgeons supported the surgical removal of the entire clitoris (28–30). This was largely based on the misunderstanding that the clitoris was unnecessary for sexual function (31).

Gross, in his 1966 publication titled “*Clitorectomy for Sexual Abnormalities: Indications and Techniques*” described the technique used in 47 patients (29).

The procedure began by placing a Foley catheter in the urethra to avoid injury. With the clitoris placed on stretch, the base was “severed circumferentially, using an elliptical incision” (29). The dorsal vein and suspensory ligaments were then divided. The corpora cavernosa were subsequently dissected proximally to the bifurcation of the crura and each of the crura was then separated from its attachment to the inferior aspect of the ischial rami. Prior to complete removal, a hemostat was carefully placed across the attenuated tip of the corpus to control the clitoral artery. Unlike previous authors who had advocated partial clitoral resection, Gross emphasized the complete removal of all tissues in order to eliminate the painful neuromata, which were reported to occasionally occur in the stump after amputation. A vulvoplasty would be subsequently performed with the urogenital sinus tissue. In the results section, the authors stated, “One patient had a post-operative hematoma requiring drainage.” And concluded that “the morbidity is less than might be expected” (29).

In defense of this practice Gross stated, “Some persons have been reluctant to advocate excision of even the most grotesquely enlarged clitoris. This view apparently stems from the belief that the clitoris is necessary for normal sexual function.” He goes on to say “Such opinion is no longer tenable; there are a number of sound studies which demonstrate normal sexual response in females who have undergone clitoral extirpation.” To support this view he describes “the custom of a number of African tribes to excise the clitoris and other parts of the external genitals at pubertal ceremonies. Yet normal sexual function is observed in these females” (29).

This contention was later brought into question as reports of patients with sexual inhibition and ambivalence toward sexual activity began to surface (32).

#### Reduction Clitoroplasty

Any surgical procedure involving the clitoris carries the risk of disruption of the nerve supply (16, 33) Techniques to reduce clitoral size have attempted to minimize this risk by preserving the dorsal neurovascular bundles (34–37). There is no unanimous consensus as to the best technique for achieving this goal.

##### Complete Corpora Cavernosa Excision

Excision of the corporal bodies with preservation of the glans clitoris and attached ventral mucosa was initially described by Goodwin and later expounded upon by others (32, 38–41). In the earliest version of this operation, an incision was made at the dorsal base of the clitoris. The crura were dissected back to the bifurcation of the crura. The neurovascular bundles were then dissected off of the shaft and the crura were separately mobilized, ligated, and divided. The corporal shaft of the clitoris was then dissected distally to the glans and the corpora excised. The preserved glans and attached ventral mucosa were the recessed beneath the pubic arch (42). If a vaginoplasty was to be undertaken simultaneously, then it was generally performed using a cut-back procedure or a Fortunoff flap (43).

Improved understanding of the importance of the dorsal neurovascular bundle for clitoral innervation led Goodwin to modify his technique of reduction clitoroplasty by approaching the dissection from the ventral aspect of the clitoris (44). He felt that “preservation of the (neurovascular) bundle theoretically should preserve the sensory function of the glans clitoris” and that if the phallus could be approached ventrally potential injury to the neurovascular bundle may be lessened. In his modification, a transverse or longitudinal incision was made on the ventral aspect of the skin overlying the clitoris. The dorsal aspect of the clitoris with its associated skin and neurovascular bundles was left untouched. The shaft of the clitoris was then freed by dissecting along Buck’s fascia until the corporal bodies had been circumferentially mobilized. The shaft was then everted out of the incision and partial amputation was accomplished. The glans clitoris and the proximal stump of the corporal bodies where then sutured together.

Additional efforts to improve upon this technique ensued as surgeons found that degloving of the clitoral shaft provided improved exposure of the neurovascular bundles and hence decreased the chance of their injury.

**Figure 1:**
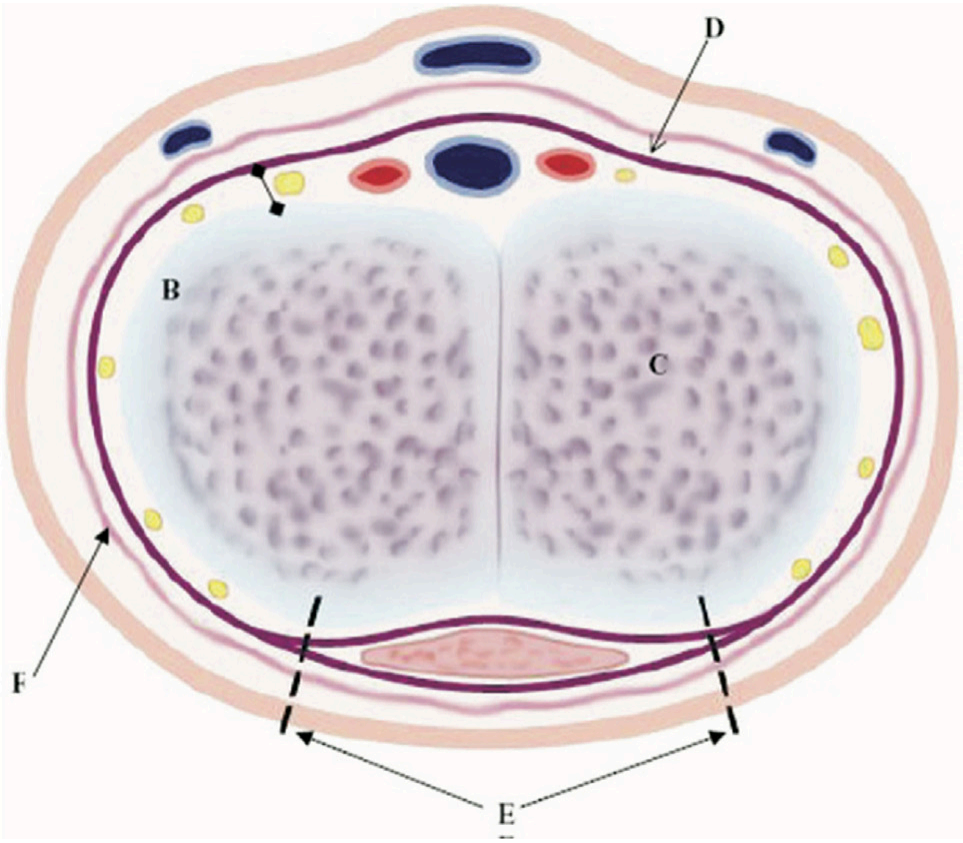
Ventral nerve sparing clitoroplasty: anatomical diagram of cross-section of clitoral shaft. NVB with nerves (yellow areas), arteries (red areas), and veins (blue areas), which are between Buck’s fascia (D) and tunica albuginea (B), corpora cavernosa tissue (C), initial ventral points of incision to begin elevation of NVB and Buck’s fascia off of tunica albuginea (E), and vascular dartos layer (F) [From Poppas et al. (37)].

**Figure 2:**
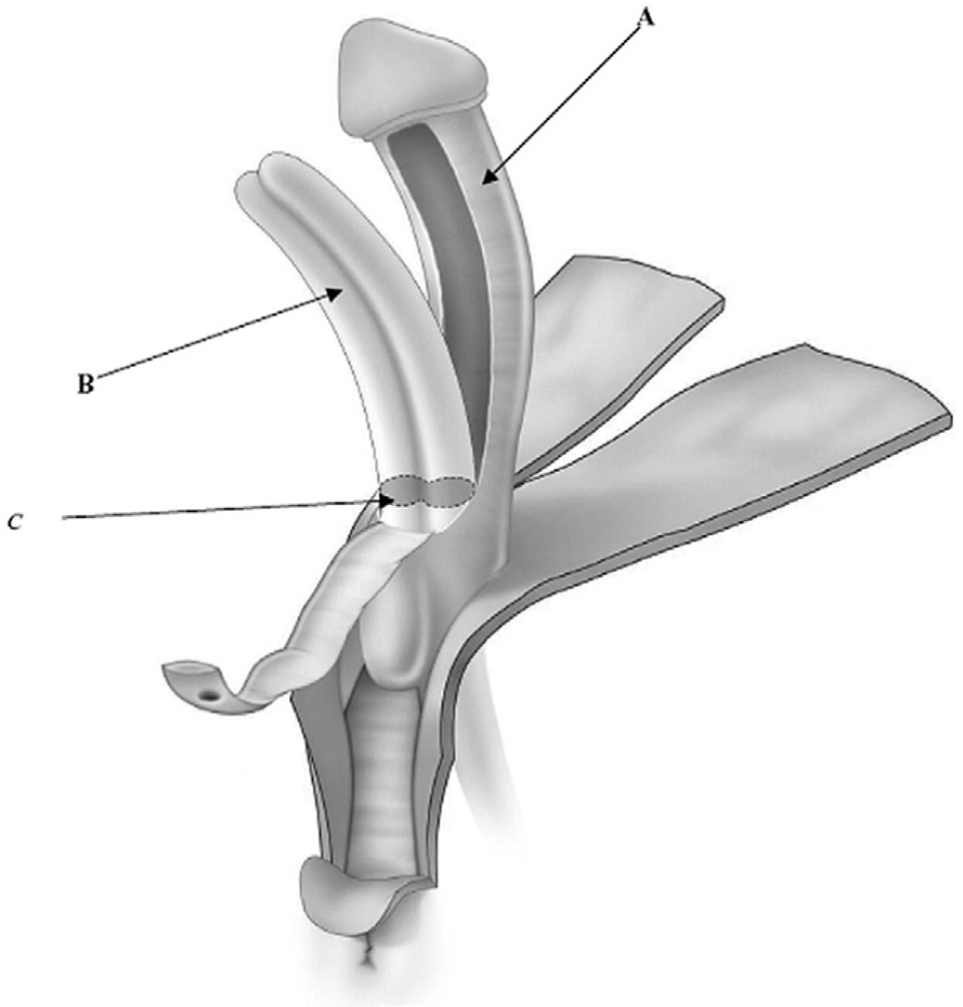
Ventral nerve sparing clitoroplasty: anatomical drawing of clitoroplasty. (A) Buck’s fascia and NVB elevated off of tunica albuginea, (B) tunica albuginea containing corpora cavernosa, and (C) point of transection of corpora cavernosa 1.5–2 cm distal to bifurcation [From Poppas et al. (37)].

The nerve-sparing ventral clitoroplasty (NSVC) as popularized by Poppas et al. begins with performing a subcoronal circumferential incision 0.5–1.0 cm proximal to the coronal margin of the glans clitoris. Care is taken not to violate Buck’s fascia as the clitoris is degloved. Dissection of the erectile bodies is facilitated by transection of the ventral plate and carried down to the crural bifurcation. Buck’s fascia is then opened using two parallel incisions lateral to the ventral midline in the vertical midline (Figure 1). All tissues external to the tunica albuginea are carefully elevated. A dilute solution of papaverine (1:100,000) is used to irrigate the tissue in order to prevent vasospasm, thrombosis, and ischemia of the neurovascular tissue. In a select number of cases, intraoperative optical coherence tomography was used to further enhance visualization of dorsal nerves *in situ*. The glans tissue is then dissected off of the corpora cavernosal bodies, leaving it supported by the tissue within Buck’s fascia (Figure 2).

The corporal bodies are then transected 1.5–2.0 cm distal to their bifurcation. Opinion differs as to how the corporal tissue proximal to the bifurcation should be managed. Many surgeons feel that maintaining this tissue and allowing for engorgement are beneficial for sexual arousal. In contrast, others feel that the remaining tissue may cause discomfort when engorged. De Jong et al. (45) incorporated the additional step of dilating the proximal corporal stumps with a metal sound to ablate the remaining proximal corporal tissues. The transected proximal ends of the corporal bodies are then oversewn using running 3-zero polydioxanone suture. The glans is then secured to the proximal ends of the corporal bodies taking great care to exclude the dorsal aspect to avoid injuring the nerves.

To document the degree to which they had preserved the neural structures of the clitoris, immunoperoxidase staining was performed with antibodies to neurofilament. In 4 of 27 patients, no dorsal nerve branches were visualized in excised erectile tissue. In 18 patients, 10 or fewer branches were found. Overall, 92% of dorsal nerves detected were 90 mm or less. The authors concluded that the scarcity of large dorsal nerves in histological specimens likely reflected satisfactory nerve preservation (37).

#### Tunica Albuginea-Sparing Clitoroplasty

While preservation of the neurovascular bundles represented a significant advance over complete clitoral ablation, concerns remain that separating these delicate structures off of the Tunica Albuginea may leave them unsupported and hence at risk of injury. To this end, techniques have been developed to preserve the Tunica Albuginea, so that the nerves and microvessels to the glans clitoris do not have to be disturbed by dissection and can benefit from the support of this scaffold.

##### Kogan Subtunical Reduction Clitoroplasty

In response to reports that permanent glans atrophy could occur when subtotal or total corpora cavernosa resection was performed (34), Kogan et al. (36) proposed a subtunical reduction of the cavernosal erectile tissue while preserving the entire tunica albuginea. A deliberate vertical incision is made through Buck’s fascia and the tunica albuginea of the shaft, exposing the hypertrophied cavernous tissue. The neurovascular bundles thus remain completely untouched in this dissection.

It is our very strong opinion that Kogan’s concept of leaving the entire tunica albuginea intact is the most logical approach yet suggested for clitoroplasty. This technique eliminates dissection of the neurovascular bundles and provides excellent support of the neurovascular complex. We describe our modification of the Kogan technique below.

Appropriate preoperative stress steroid dosing is given under the direction of our pediatric endocrinologist. We feel that proper visualization and mobilization of tissues can be performed with the patient in the supine position or the well-padded lithotomy position. If supine positioning is chosen, the patient undergoes full circumferential body prep from the nipples down.

**Figure 3:**
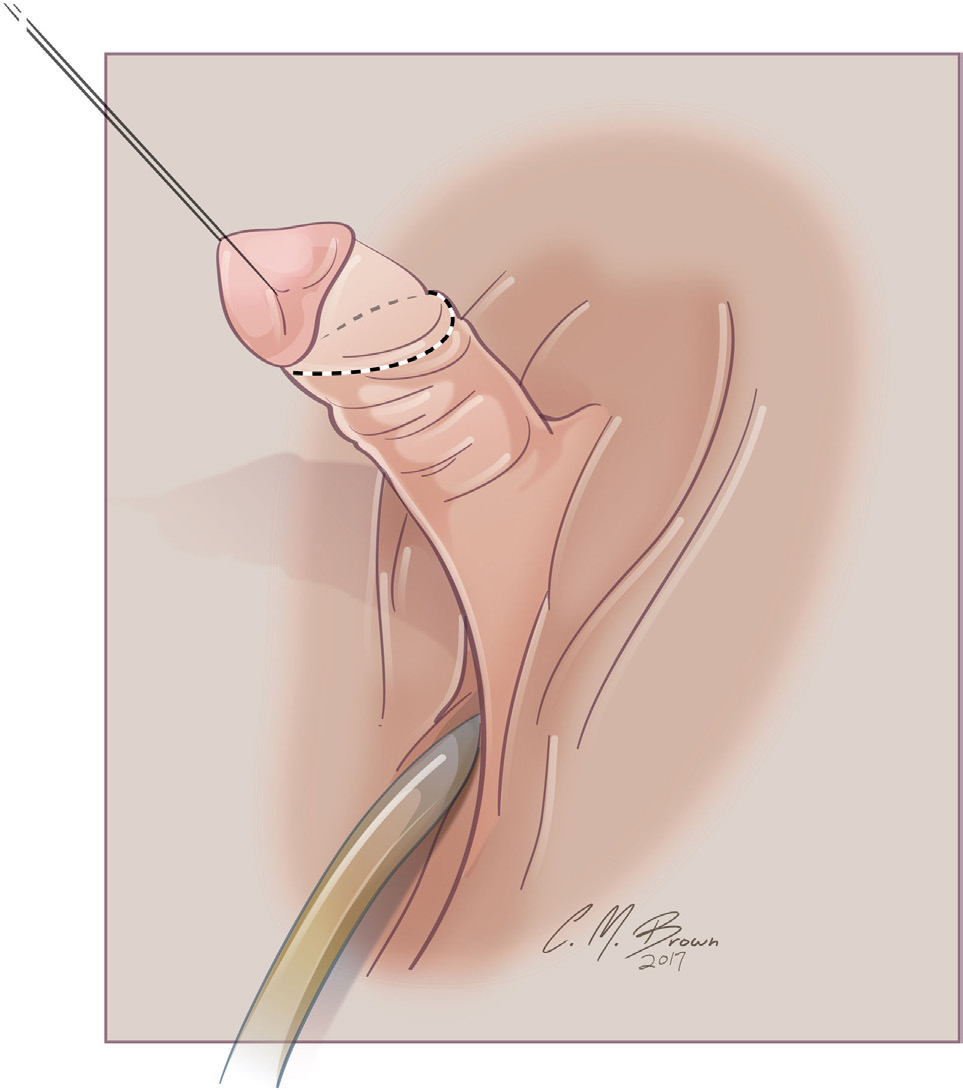
Albuginea sparing clitoroplasty: lines of skin incision. When degloving the clitoris, special attention is taken to leave the entire inner preputial layer of skin of at the coronal margin along the dorsal aspect of the phallus. The incision is therefore carried out approximately 1 cm or more proximal to the coronal margin. This cuff of skin is highly sensitive, second only to the glans itself.

A stay suture is placed vertically in the glans clitoris, and a skin scribe is used to mark out the lines of incision (Figure 3). When degloving the clitoris, special attention is taken to leave the entire inner preputial layer of skin of at the coronal margin along the dorsal aspect of the phallus. The incision is therefore carried out approximately 1 cm or more proximal to the coronal margin. This cuff of skin is highly sensitive, second only to the glans itself (46). This tissue will later be used to create the inner aspect of a classic tent like morphology for the clitoral hood. The clitoris is degloved and the ventral plate of tissue divided just under the glans clitoris. Great care must be taken to avoid injury to the underlying neurovascular structures since the plane between the clitoral skin and the neurovascular bundles may often be less well defined than it is with the penis.

**Figure 4:**
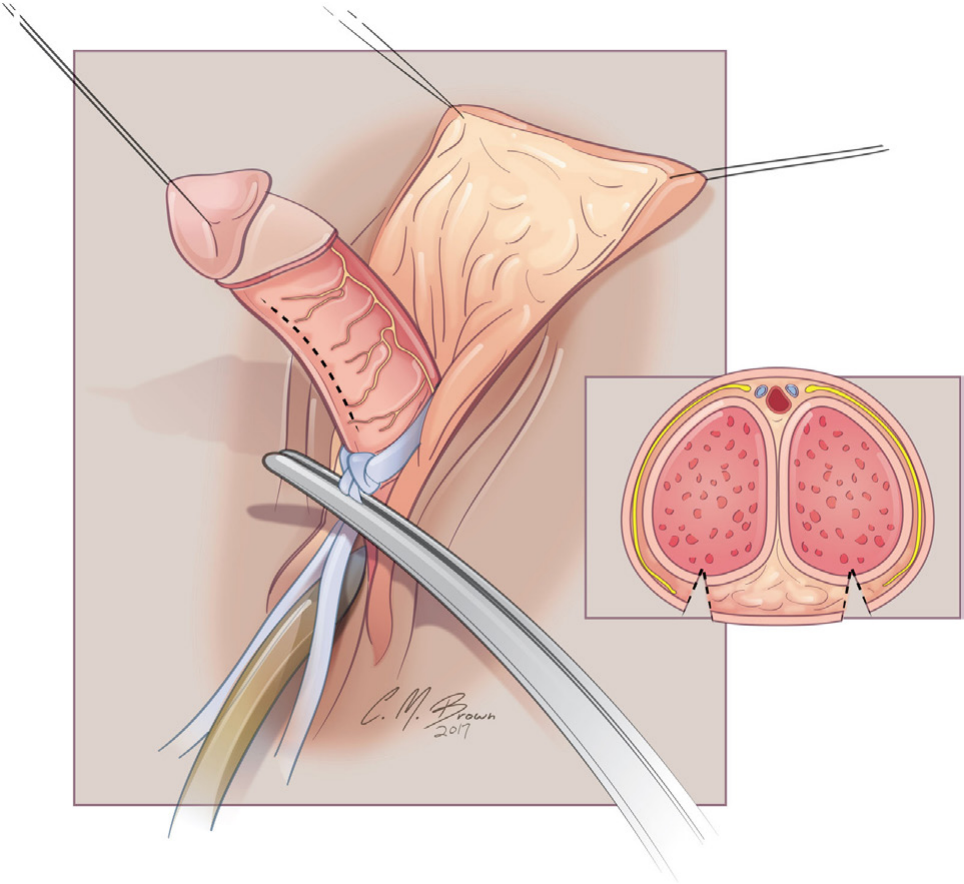
Albuginea sparing clitoroplasty: excision to remove corpora cavernosal tissue. Left lateral view demonstrating ventral location of incision into tunica albuginea. Inset demonstrates two parallel incisions on ventral aspect of shaft.

**Figure 5:**
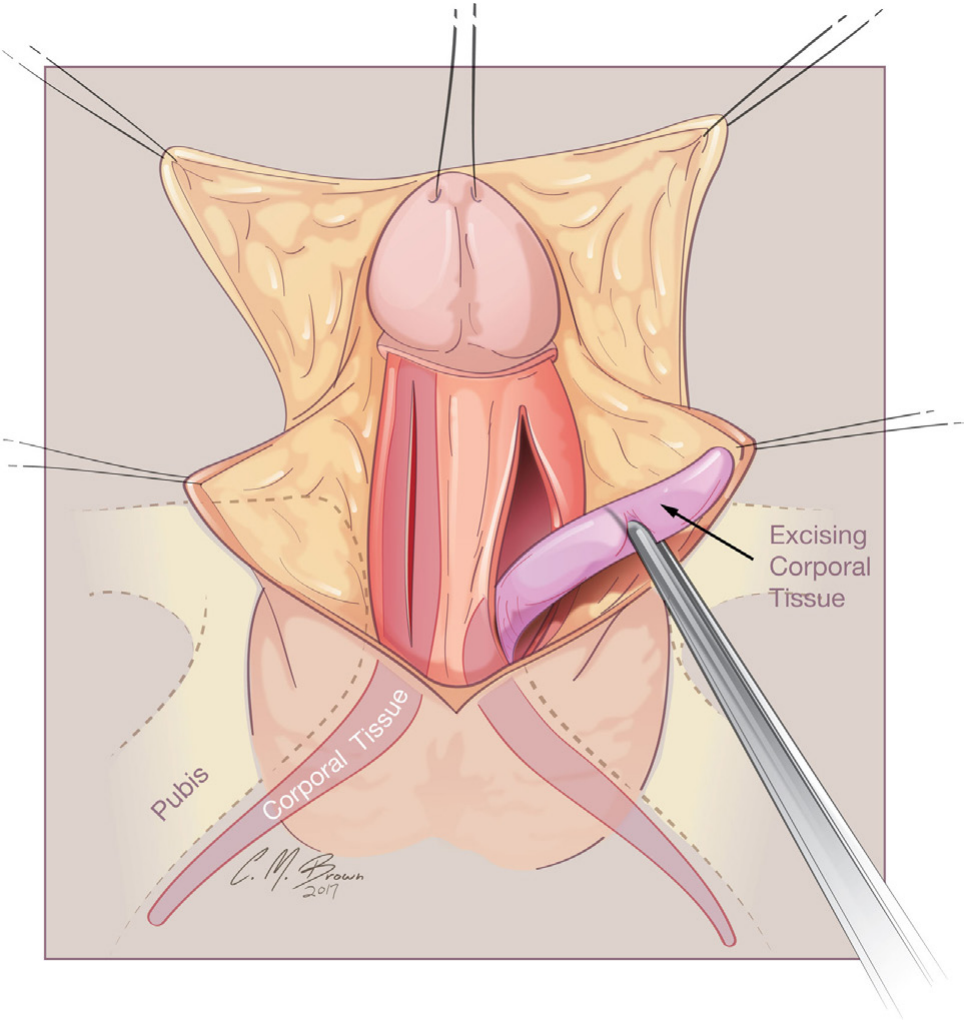
Albuginea sparing clitoroplasty: corporal tissue being shelled out of preserved tunica albuginea.

**Figure 6:**
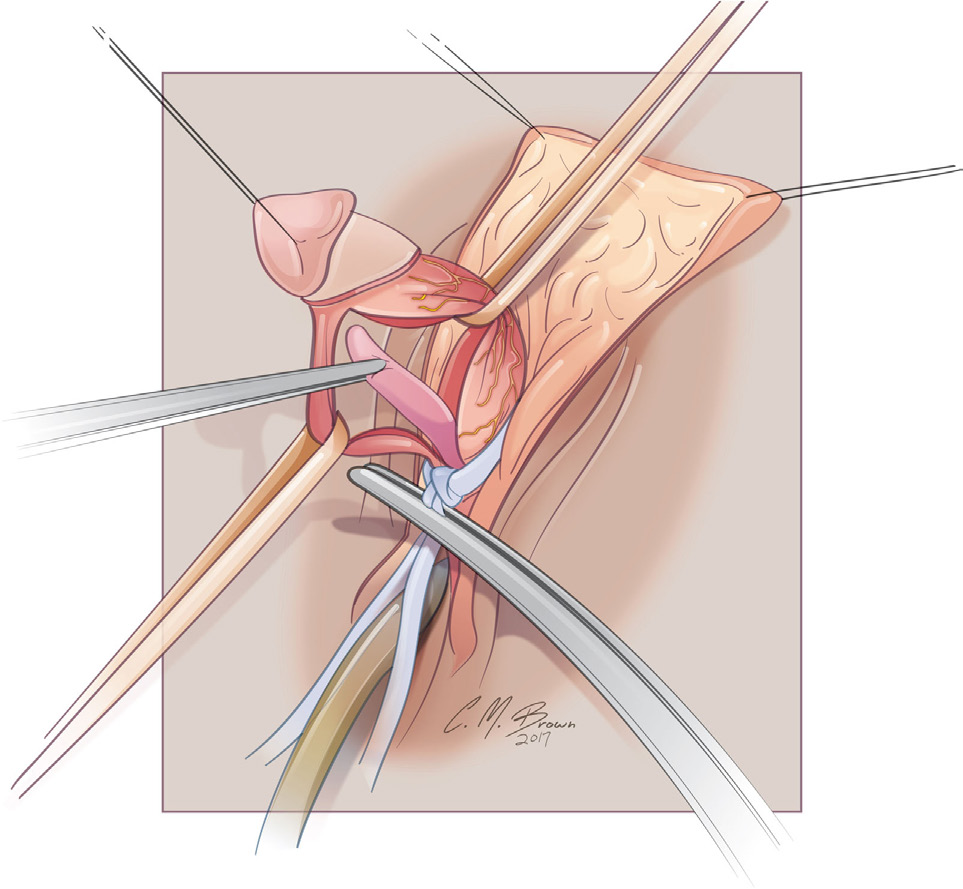
Albuginea sparing clitoroplasty: lateral view of corporal tissue dissected free from tunica albuginea. Note septum distracted anteriorly—this will be excised prior to folding of the tunica albuginea into cranial location under the mons pubis.

**Figure 7:**
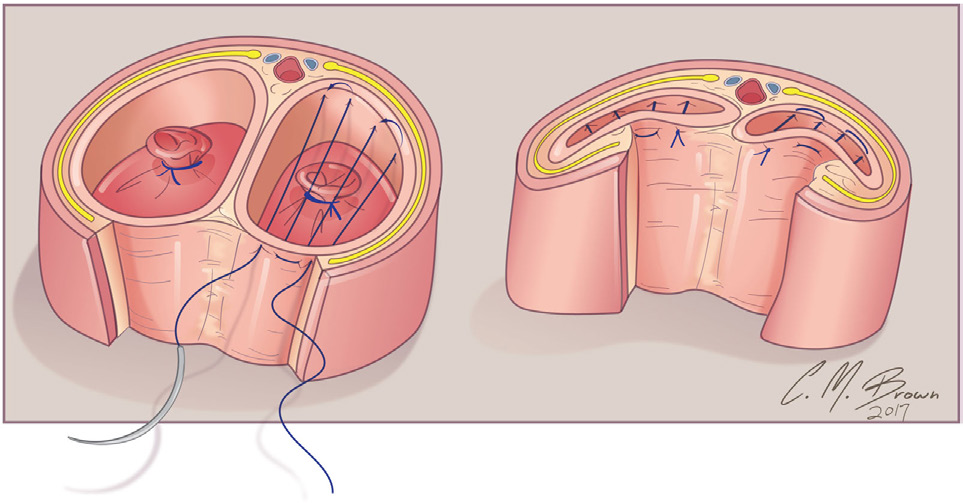
Albuginea sparing clitoroplasty: cross section of corpora cavernosa just distal to crural bifurcation. The proximal extent of the erectile tissue has been controlled bilaterally with 5-0 PDS ties. To provide proximal hemostasis, the ventral layer of the tunica albuginea is sutured to the inner layer of the dorsal aspect of the tunica albuginea just distal to the bifurcation with running 5-zero polydioxanone suture. Great care must be taken in this step to avoid placing the sutures too deep into the dorsal tunica albuginea for fear of injuring the exact nerves that one is aiming to preserve.

A tourniquet is now placed at the base of the clitoris. Alternatively, an assistant can put pressure at the base of each corpora with a Kittner dissector. Kogan felt that the subsequent incisions in the tunica albuginea could be made dorsally, laterally, or ventrally “as individual local anatomy allows.” With our improved understanding of neurovascular anatomy of the clitoris, we now feel that placing paired incisions ventrally is most appropriate to minimize damage to any nerve fibers (Figures 4–6). The incisions extend from the glans to the bifurcation to expose the corpora cavernosa tissue, which is shelled out from the tunical coverings. The tunics are left undisturbed, except for the ventral incision, so as to avoid any mobilization or disturbance of the dorsal neurovascular bundle. It is frequently more difficult to shell the tissue out of the second side than out of the first. The proximal ends of the erectile tissue are suture ligated. To provide proximal hemostasis, the ventral layer of the tunica albuginea is sutured to the inner layer of the dorsal aspect of the tunica albuginea just distal to the bifurcation with running 5-zero polydioxanone suture. Great care must be taken in this step to avoid placing the sutures too deep into the dorsal tunica albuginea for fear of injuring the exact nerves that one is aiming to preserve (Figure 7).

**Figure 8:**
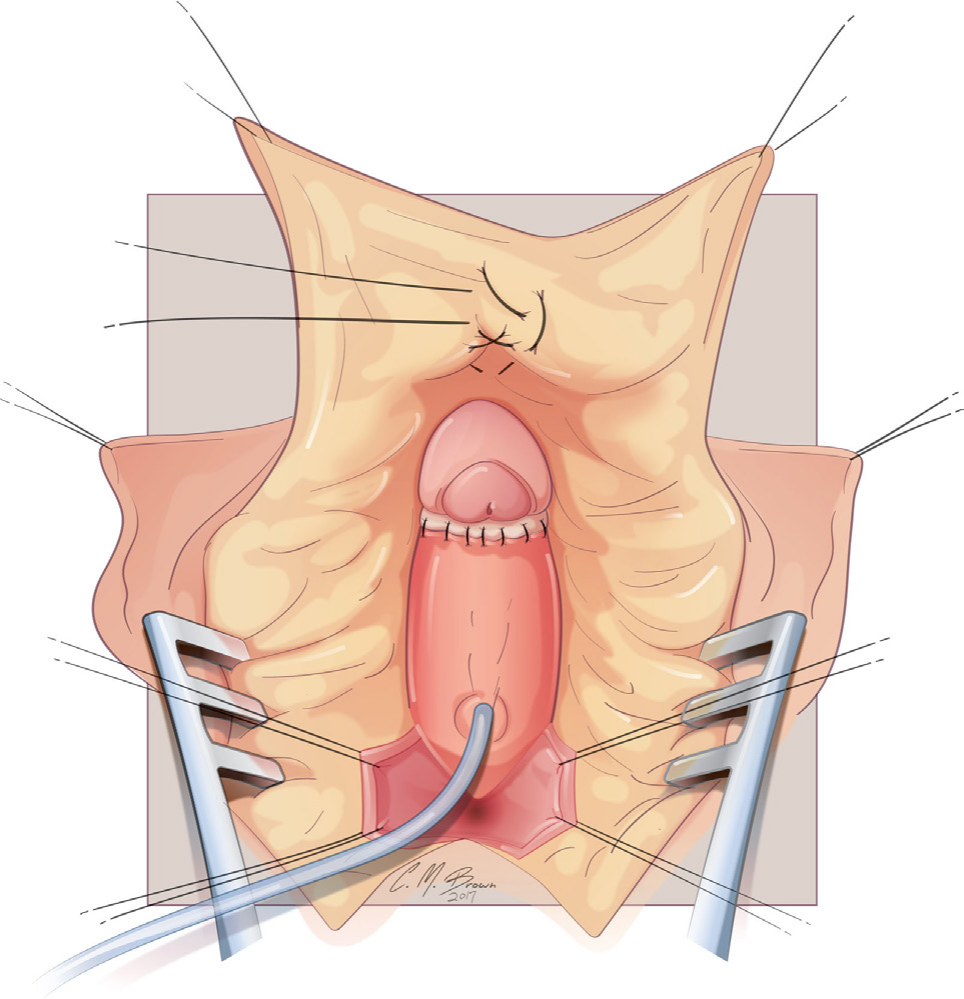
Albuginea sparing clitoroplasty: the glans clitoris sewn to the ventral aspect of the tunica albuginea at the level of the crural bifurcation. The ventral urethral plate that was reflected inferiorly for the dissection is then secured to the base of the glans.

The glans is now sewn to the ventral aspect of the tunica albuginea at the level of the crural bifurcation (Figure 8). Thereafter, the neurovascular structures with there supporting tunica albuginea are folded gently back and recessed under the mons pubis. The most superior aspect of the tissue (at the point where it is folded) is then carefully secured to the pubis symphysis with two 3-zero polypropylene sutures. In cases of CAH, the vaginoplasty is subsequently performed. Upon completion of the vaginoplasty, the dorsal skin of the clitoris is split longitudinally and the flaps utilized for construction of the labia minora. A clitoral hood is constructed from the intact tissue of the dorsal shaft skin and then secured to the preserved inner layer of prepucial skin that had been left with the glans during the early part of the dissection.

We have utilized this technique in over 80 cases during the past 10 years and have noted no glans atrophy.

##### Girth-Reduction Clitoroplasty

**Figure 9:**
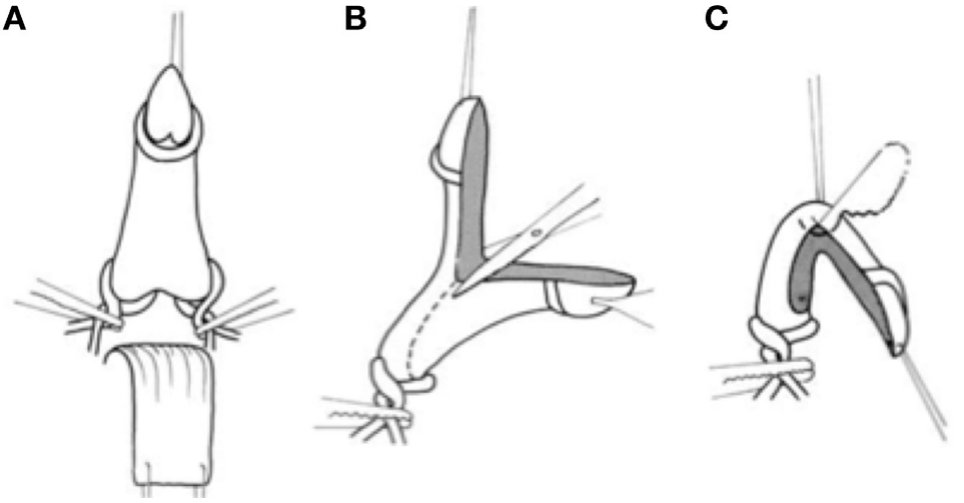
Girth reduction clitoroplasty: once the clitoris has been degloved, **(A)** vessel loops are placed at the base of each corpora in order to maintain hemostasis. **(B)** The glans and corporal bodies are then divided in the coronal plane and the ventral aspect excised and discarded. **(C)** The shaft is then folded and sutured on each side so that the raw corpora cavernosa are approximated [From Hutson et al. (47)].

One additional technique that serves to preserve the tunica albuginea is the Girth-reduction clitoroplasty. This procedure was first devised by Robert Fowler in the early 1970s and later popularized by Hutson et al. (47). The initial degloving of the clitoris, release of the ventral plate, and identification of the corporal bifurcation are similar to that described above. The authors then utilize a right-angle dissector to dissect between the crural bifurcation. Vascular loops are passed around each crura to act as tourniquets. The glans and corporal bodies are then divided in the coronal plane and the ventral aspect excised and discarded (Figure 9). Great care is taken not to encroach on the dorsal half of the circumference, thereby protecting the neurovascular bundles that remain adherent to the tunica albuginea and protected by Buck’s fascia. The shaft is then folded and sutured on each side so that the raw corpora cavernosa are approximated and only the glans is free. The glans is tublarized in the midline plane to reconstruct it into a conical shape. The hairpin bend on the reconstructed clitoris is sutured to the symphysis.

The Girth-reduction clitoroplasty allows reduction of the clitoris without dissection of the neurovascular bundles extending between the 9 and 3 o’clock positions. A portion of the corporal tissue is maintained. The authors sight studies by O’Connell et al. and Rees et al. that demonstrate that the erectile tissue is not only related to the ventral aspect of the shaft but also runs along the lateral walls of the urethra (3, 48–50). They feel that this tissue is responsible for the rigidity of the vaginal wall during orgasm and that a portion of the erectile tissue should be preserved during surgery for clitoral reduction.

#### Corporal-Sparing Techniques (Clitoral Recession/Corporal Preservation)

In the early 1960s, procedures to recess the intact clitoris were devised. While preserving all erectile tissue, these procedures may be technically challenging in cases where the clitoris is significantly enlarged. In the past decade, Pippi Salle has devised an additional novel technique aimed at preserving all component structures of the clitoris with the expressed aim of having the erectile tissue available to reconstruct the phallus should the patient elect for such a choice at a later date.

##### Latimer Tunneling

In 1961, Latimer proposed a procedure to replace clitorectomy. In this technique, the skin of the phallus was removed, the suspensory ligament divided, and the corpora freed up for some distance from their attachment to the pubis. The clitoral head was often reduced in size by excising a wedge of tissue from the front of the glans. The intact corporal bodies and modified glans were then tunneled down to its new position at the top of the vestibule just above the urethral meatus. The labia majora were then brought together in the midline above the introitus to close the defect where the clitoral shaft skin had been removed (51). It was Lattimer’s opinion that the technique was most appropriate for patients with a moderately enlarged clitoris.

He reported satisfactory cosmetic results in his first 11 cases. The long-term success of this operation cannot be determined since extended follow-up of patients undergoing this procedure has not been reported.

##### Randolph Recession

In 1970, Randolph et al. described a modification of Latimer’s surgical technique that also sought to preserve all of the corporal components. The procedure consisted of thorough dissection of the corporal bodies down to their most proximal attachments to the pubis (as if preparing for clitoral removal). The clitoris would then be recessed under the pubis by placing sutures in the dorsum of the corporeal fascia near the glans and securing them to the periosteum of the inferior margin of the symphysis (52, 53). As a result, the corporal bodies would not be as readily palpable. The authors published the functional results in patients undergoing their procedure as infants (54). The median age of the 9 patients available for interview was 21 years. It was reported that eight of the nine patients achieved regular orgasms. The authors stated that it was their belief that preservation of all clitoral tissue was highly desirable in “achieving the most nearly normal sexual response.” They surmised that eliminating the ability to achieve any form of erection might significantly handicap orgasm.

The potential disadvantage of retaining intact corporal bodies is the possible discomfort that they may cause when fully engorged. One of their patients did complain of pain with erection on sexual excitation. Allen et al. (42) reported on six patients who had been treated by clitoral recession and found that several had pain with erection. This led them to advocate for excision of the corporal bodies.

##### Pippi Salle Corporal-Sparing Dismembered Clitoroplasty

**Figure 10:**
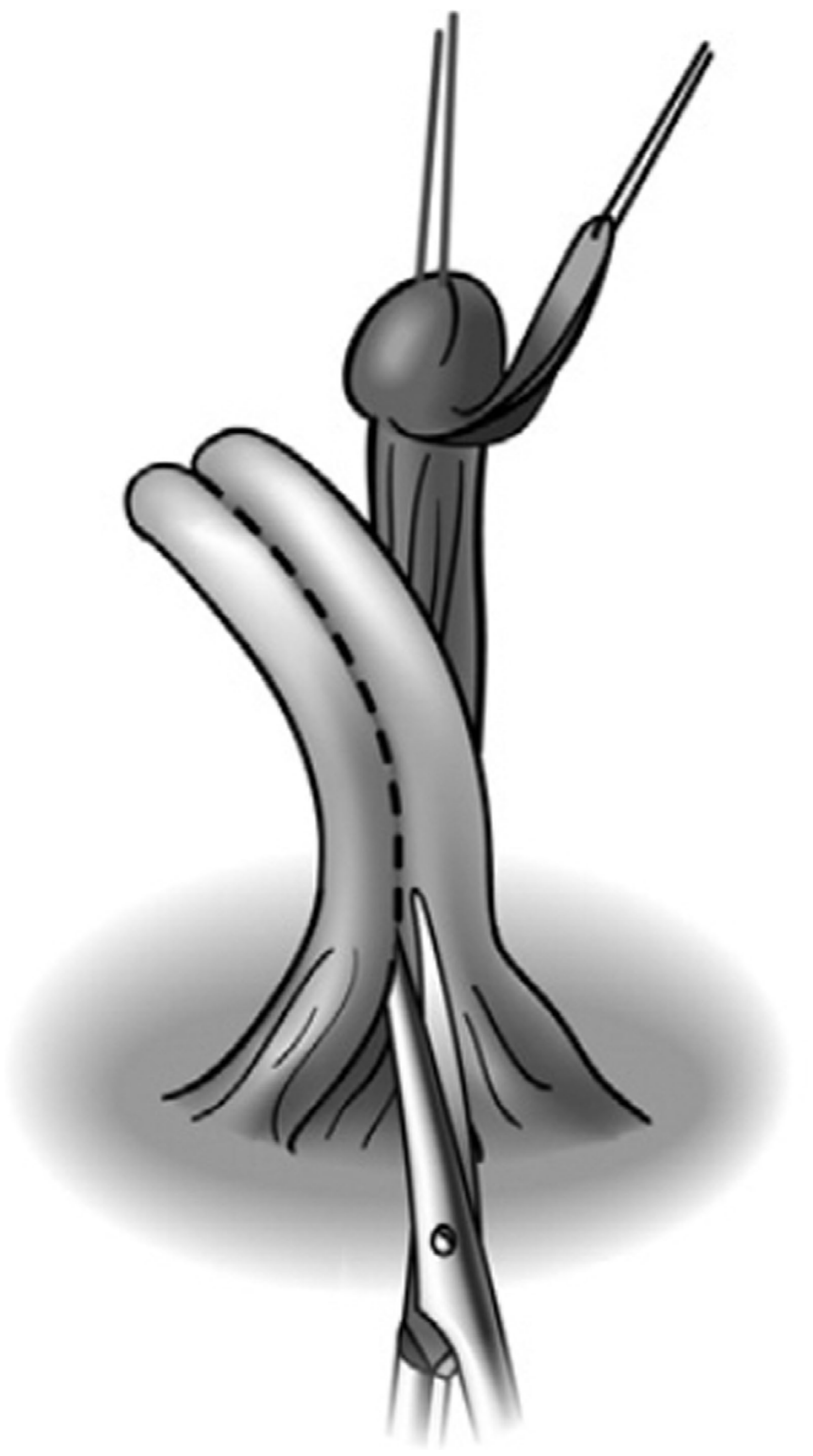
Corporal sparing dismembered clitoroplasty: neurovascular bundles and glans with attached urethral plate dissected free from paired corpora cavernosa [From Pippi Salle et al. (55)].

**Figure 11:**
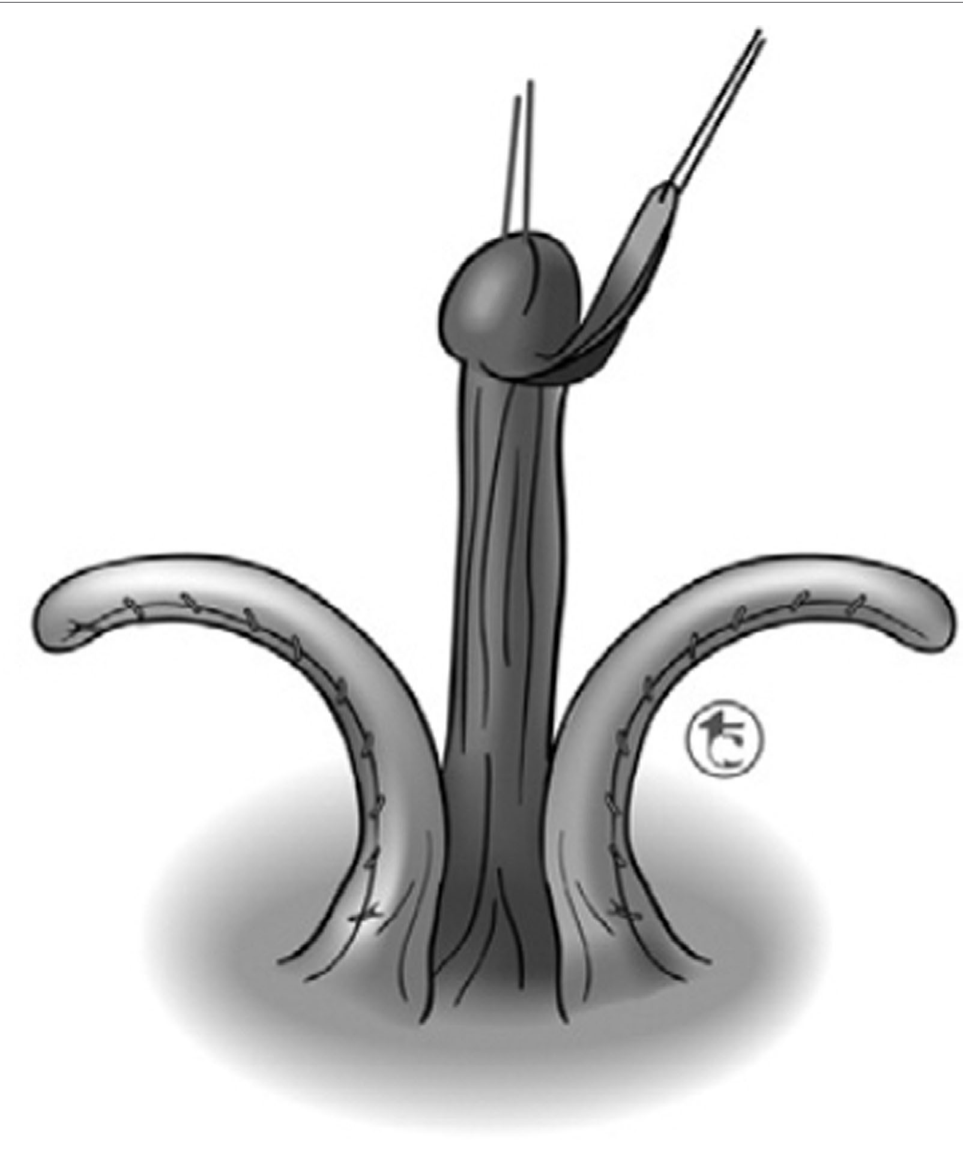
Corporal sparing dismembered clitoroplasty: once the bifurcation has been exposed, the corpora are divided sharply beginning caudally and progressing distally. The edges of the opened tunica albuginea are then approximated with fine absorbable suture to maintain hemostasis [From Pippi Salle et al. (55)].

**Figure 12:**
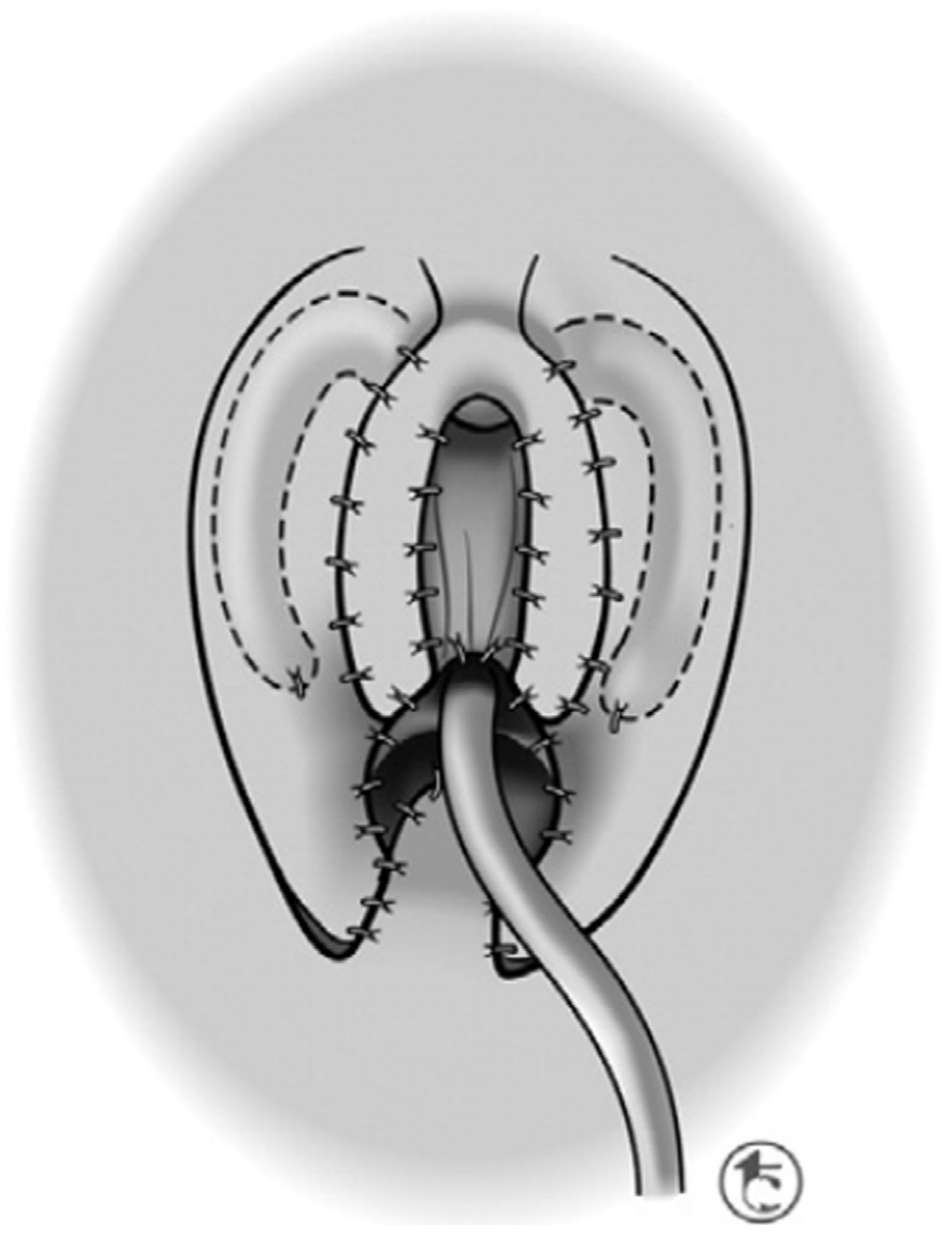
Corporal sparing dismembered clitoroplasty: each corporal body is rotated inferolaterally and placed in a dartos pouch in the ipsilateral labia majora [From Pippi Salle et al. (55)].

Recognizing that irreversibility continues to be the principle issue that concerns surgeons and creates parental anxiety, Pippi Salle et al. (55, 56) have developed a corporal-sparing clitoroplasty with the goal of preserving all original structures. The clitoris is degloved as described in the previous section. The neurovascular bundles are then dissected free from the corpora starting as ventrally as possible. The urethral plate is divided just distal to the opening of the urogenital sinus and maintained with the glans, which is dissected free from the distal ends of the corpora cavernosa (Figure 10). The urogenital sinus itself is freed from the anterior corporal surface and reflected caudally. Once the bifurcation has been exposed, the corpora are divided sharply beginning caudally and progressing distally (Figure 11). The edges of the opened tunica albuginea are then approximated with fine absorbable suture. Each hemicorpora is then rotated laterally and inferiorly and placed in a dartos pouch in each labia majora (Figure 12). Glans reduction is performed by superficial excision of the epithelium of the glanular groove and secured to the pubic bone approximately 1 cm above the original bifurcation of the corpora. The authors have found the cosmetic appearance to be quite satisfactory.

The procedure, which was initially described in eight patients, provided a satisfactory cosmetic result. The authors note that up to 5% of patients with CAH may have gender dysphoria later in life and that their procedure provides a potential for surgical transition back to an intact phallus. Ultimately, the success of this operation will depend on long-term follow-up and the demonstration of intact sensation and sexual function.

#### Complications

##### Postoperative Pain

Clitoral pain or enlargement can occur after clitoral recession or incomplete reduction. This is perhaps especially true in patients with CAH, who due to the risk of inadequate suppression during their lifetime, have the potential to experience additional growth of the clitoral tissue. Newman et al. (54) in reporting on their series of clitoral recession noted that two of their four patients with CAH experienced pain with arousal or intercourse. When this does occur, extensive counseling should be undertaken to assure that the patient understands the risks of reoperation. Prior to surgically addressing this issue, it is imperative that adequate androgen suppression is accomplished. Reifsnyder et al. (57) have shown in a series of six female patients (mean 22 ± 8 years) that nerve-sparing clitoroplasty can be successfully performed to address this issue.

##### Vascular Injury

Clitoral atrophy has been documented to occur in patients undergoing clitoroplasty. Mollard et al. (34) reported on the potential complication of glans atrophy following subtotal or total shaft resection. Alizai et al. (58) in their report of 14 children at a mean age of 13 years found that 4 had experienced atrophy of the glans clitoris. This finding underscores the importance of safeguarding the vascularity of the glans. Many authors have emphasized the importance of minimizing traction on the neurovascular bundle so as to minimize vasospasm (59). Boccardi et al. reported no evidence of glans atrophy using the Passerini technique (ESPU London, 1996).

##### Neurologic Status

The most frequently discussed concern with regard to clitoral surgery is the potential for neurologic injury. These concerns have led various investigators to utilize intraoperative imaging of the nerves as well as measurement of pudendal evoked potentials to demonstrate that the electromyographic responses are not significantly compromised (37, 60). Although clitoroplasty with preservation of the dorsal nerves can lead to normal sensation and orgasmic potential, this is not always the case (15, 61, 62). Despite what would appear to be a clear advantage of neurovascular preservation over total excision, only one out of every three women who have undergone a nerve-sparing procedure report sensitivity to temperature and vibration that is similar to unaffected women (15).

#### Psychosocial Outcomes

Overall favorable quality of life and good psychological health have been reported in CAH patients (63–65). A study of 45 CAH women revealed high quality of life comparable to age- and education-matched controls (64). However, CAH patients were more often single (66.7 vs. 47.8%); were less sexually active, displayed more negative body image; had more negative self-image with regard to self-confidence, sociability, and social acceptance. Despite these findings, good physical function, active coping mechanism, and high global satisfaction with life resulted in overall high quality of life. Women with the salt-losing form usually have worse psychosexual functioning than those with the non-salt-losing form (66).

#### Timing of Treatment

There is no unanimous opinion as to the optimal timing for clitoroplasty. Proponents of early surgery point to adult patients who have undergone feminizing surgery at various ages who feel early surgery to be favorable to late surgery (67). Girls who have undergone surgery have a satisfactory cosmetic outcome, as assessed by health care providers and a good quality of life and a low incidence of gender dysphoria as reported by their parents (68, 69). Proponents of late surgery rightfully point out that postponing surgery to a later age allows the individual to feel more empowered in that they are able to participate in the decision process. Although it can be surmised that the psychological impact of late genital surgery is likely to be more significant than in early life, there is no definitive evidence to support whether early or late surgery is better (61, 70, 71).

## Conclusion

The ideal treatment for the enlarged clitoris, whether it be an observation or surgical management, is to be made on an individual basis in the context of a multidisciplinary team. Many surgical techniques have been described, with the underlying condition of the child influencing which choice may be best suited in each individual case. Additional outcomes based research will help to inform both physicians and their patients as to the results that can be anticipated from these procedures.

## Author Contributions

Both authors have contributed in a substantial material fashion to this manuscript. Both have written the manuscript and helped design the new graphics to describe the technique which the authors advocate.

## Conflict of Interest Statement

The authors declare that the research was conducted in the absence of any commercial or financial relationships that could be construed as a potential conflict of interest.

## References

[1] Schnitzer JJ Donahoe PK. Surgical treatment of congenital adrenal hyperplasia. Endocrinol Metab Clin North Am (2001) 30:137. 10.1016/S0889-8529(08)70023-9 11344932

[2] Baskin LS Erol A Li YW Liu WH Kurzrock E Cunha GR. Anatomical studies of the human clitoris. J Urol (1999) 162:1015. 10.1097/00005392-199909000-00014 10458423

[3] O’Connell HE Sanjeevan KV Hutson JM. Anatomy of the clitoris. J Urol (2005) 174:1189. 10.1097/01.ju.0000173639.38898.cd 16145367

[4] Brodie KE Grantham EC Huguelet PS Caldwell BT Westfall NJ Wilcox DT Study of clitoral hood anatomy in the pediatric population. J Pediatr Urol (2016) 12:177.e1 10.1016/j.jpurol.2015.12.006 26851151

[5] Therrell BL. Newborn screening for congenital adrenal hyperplasia. Endocrinol Metab Clin North Am (2001) 30:15. 10.1016/S0889-8529(08)70017-3 11344933

[6] Brentnall CP Case of arrhenoblastoma complicating pregnancy. J Obstet Gynaecol Br Emp (1945) 52:235 10.1111/j.1471-0528.1945.tb07622.x

[7] Persechini ML Motton S Leguevaque P Donadille F Escourrou G Vierasu B Virilising ovarian tumour: a case associating a Sertoli-Leydig cell tumour and a Brenner tumour. Gynecol Endocrinol (2011) 27:345. 10.3109/09513590.2010.492883 20569103

[8] Kunz GJ Klein KO Clemons RD Gottschalk ME Jones KL. Virilization of young children after topical androgen use by their parents. Pediatrics (2004) 114:282. 10.1542/peds.114.1.282 15231947

[9] Haddad NG Vance GH Eugster EA Davis MM Kaefer M Turner syndrome (45x) with clitoromegaly. J Urol (2003) 170:1355 10.1097/01.ju.0000085983.81063.3f 14501769

[10] Kaefer M 45,X/46,XY mosaicism: a spectrum of phenotypic expression and management. Dialog Pediatr Urol (2000) 23:6.

[11] Creighton S Chernausek SD Romao R Ransley P Salle JP Timing and nature of reconstructive surgery for disorders of sex development – introduction. J Pediatr Urol (2012) 8:602 10.1016/j.jpurol.2012.10.001 23146296

[12] Kearse WS Jr Ritchey ML Clitoral enlargement secondary to neurofibromatosis. Clin Pediatr (Phila) (1993) 32:303 10.1177/000992289303200511 8324976

[13] Williams CE Nakhal RS Achermann JC Creighton SM. Persistent unexplained congenital clitoromegaly in females born extremely prematurely. J Pediatr Urol (2013) 9:962. 10.1016/j.jpurol.2013.03.001 23619354 PMC3857598

[14] Greaves R Hunt RW Zacharin M. Transient anomalies in genital appearance in some extremely preterm female infants may be the result of foetal programming causing a surge in LH and the over activation of the pituitary-gonadal axis. Clin Endocrinol (Oxf) (2008) 69:763. 10.1111/j.1365-2265.2008.03298.x 18466346

[15] Crouch NS Liao LM Woodhouse CR Conway GS Creighton SM. Sexual function and genital sensitivity following feminizing genitoplasty for congenital adrenal hyperplasia. J Urol (2008) 179:634. 10.1016/j.juro.2007.09.079 18082214

[16] Crouch NS Minto CL Laio LM Woodhouse CR Creighton SM. Genital sensation after feminizing genitoplasty for congenital adrenal hyperplasia: a pilot study. BJU Int (2004) 93:135. 10.1111/j.1464-410X.2004.04572.x 14678385

[17] Warne G Grover S Hutson J Sinclair A Metcalfe S Northam E A long-term outcome study of intersex conditions. J Pediatr Endocrinol Metab (2005) 18:555. 10.1515/JPEM.2005.18.6.555 16042323

[18] Lee PA Houk CP Ahmed SF Hughes IA International Consensus Conference on Intersex Organized by the Lawson Wilkins Pediatric Endocrine Society and the European Society for Paediatric Endocrinology Consensus statement on management of intersex disorders. International Consensus Conference on Intersex. Pediatrics (2006) 118:e488 10.1542/peds.2006-0738 16882788

[19] Speiser PW Azziz R Baskin LS Ghizzoni L Hensle TW Merke DP A summary of the Endocrine Society Clinical Practice Guidelines on congenital adrenal hyperplasia due to steroid 21-hydroxylase deficiency. Int J Pediatr Endocrinol (2010) 2010:494173. 10.1155/2010/494173 20981249 PMC2963799

[20] Rink RC Kaefer M Surgical management of intersexuality, cloacal malformation, and other abnormalities of the genitalia in girls. In: Wein AJ Kavoussi LR Novick AC Partin AW Peters CA, editors. Campbell’s Urology. 10th ed. Philadelphia, PA: W.B. Saunders (2011).

[21] Merke DP Bornstein SR. Congenital adrenal hyperplasia. Lancet (2005) 365:2125. 10.1016/S0140-6736(05)66736-0 15964450

[22] Jääskeläinen J Levo A Voutilainen R Partanen J. Population-wide evaluation of disease manifestation in relation to molecular genotype in steroid 21-hydroxylase (CYP21) deficiency: good correlation in a well defined population. J Clin Endocrinol Metab (1997) 82:3293. 10.1210/jc.82.10.3293 9329356

[23] Joint LWPES/ESPE CAH Working Group. Consensus statement on 21-hydroxylase deficiency from the Lawson Wilkins Pediatric Endocrine Society and the European Society for Paediatric Endocrinology. J Clin Endocrinol Metab (2002) 87:4048 10.1210/jc.2002-020611 12213842

[24] Bonfig W Bechtold S Schmidt H Knorr D Schwarz HP. Reduced final height outcome in congenital adrenal hyperplasia under prednisone treatment: deceleration of growth velocity during puberty. J Clin Endocrinol Metab (2007) 92:1635. 10.1210/jc.2006-2109 17299071

[25] Merke DP. Approach to the adult with congenital adrenal hyperplasia due to 21-hydroxylase deficiency. J Clin Endocrinol Metab (2008) 93:653. 10.1210/jc.2007-2417 18326005 PMC2266964

[26] Ross RJ Rostami-Hodjegan A. Timing and type of glucocorticoid replacement in adult congenital adrenal hyperplasia. Horm Res (2005) 64(Suppl 2):67. 16286775 10.1159/000087757

[27] Schaeffer TL Tryggestad JB Mallappa A Hanna AE Krishnan S Chernausek SD An evidence-based model of multidisciplinary care for patients and families affected by classical congenital adrenal hyperplasia due to 21-hydroxylase deficiency. Int J Pediatr Endocrinol (2010) 2010:692439. 10.1155/2010/692439 20339513 PMC2842898

[28] Hendren WH Crawford JD Adrenogenital syndrome: the anatomy of the anomaly and its repair. Some new concepts. J Pediatr Surg (1969) 4:49 10.1016/0022-3468(69)90183-3 5813593

[29] Gross RE Randolph J Crigler JF Jr Clitorectomy for sexual abnormalities: indications and technique. Surgery (1966) 59:300. 5913502

[30] Jones HW Jr Jones GE The gynecological aspects of adrenal hyperplasia and allied disorders. Am J Obstet Gynecol (1954) 68:1330. 13207225

[31] Hampson JG Hermaphroditic genital appearance, rearing and eroticicsm in hyperadrenocorticism. Bull Johns Hopkins Hosp (1955) 96:265. 14378808

[32] Mininberg DT Phalloplasty in congenital adrenal hyperplasia. J Urol (1982) 128:355. 7202053 10.1016/s0022-5347(17)52924-9

[33] Creighton SM Minto CL Steele SJ. Objective cosmetic and anatomical outcomes at adolescence of feminising surgery for ambiguous genitalia done in childhood. Lancet (2001) 358:124. 10.1016/S0140-6736(01)05343-0 11463417

[34] Mollard P Juskiewenski S Sarkissian J. Clitoroplasty in intersex: a new technique. Br J Urol (1981) 53:371. 10.1111/j.1464-410X.1981.tb03200.x 7260551

[35] Kogan SJ. Feminizing genital reconstruction for male pseudohermaphroditism. Eur J Pediatr (1993) 152(Suppl 2):S85. 10.1007/BF02125447 8339749

[36] Kogan SJ Smey P Levitt SB. Subtunical total reduction clitoroplasty: a safe modification of existing techniques. J Urol (1983) 130:746. 6887407 10.1016/s0022-5347(17)51436-6

[37] Poppas DP Hochsztein AA Baergen RN Loyd E Chen J Felsen D. Nerve sparing ventral clitoroplasty preserves dorsal nerves in congenital adrenal hyperplasia. J Urol (2007) 178:1802. 10.1016/j.juro.2007.03.186 17707008

[38] Goodwin WE Surgical revision of the enlarged clitoris. In: de la Camp HB Linder F Trede M, editors. American College of Surgeons and Deutsche Gesellschaft fur Chirurgie. Joint Meeting. New York: Srpinger-Verlag (1969).

[39] Shaw A. Subcutaneous reduction clitoroplasty. J Pediatr Surg (1977) 12:331. 10.1016/0022-3468(77)90009-4 874721

[40] Spence HM Allen TD Genital reconstruction in the female with the adrenogenital syndrome. Br J Urol (1973) 45:126 10.1111/j.1464-410X.1973.tb12128.x 4735914

[41] Hendren WH Donahoe PK. Correction of congenital abnormalities of the vagina and perineum. J Pediatr Surg (1980) 15:751. 10.1016/S0022-3468(80)80278-8 7007606

[42] Allen LE Hardy BE Churchill BM. The surgical management of the enlarged clitoris. J Urol (1982) 128:351. 7109108 10.1016/s0022-5347(17)52923-7

[43] Fortunoff S Lattimer JK Edson M Vaginoplasty technique for female pseudohermaphrodites. Surg Gynecol Obstet (1964) 118:545. 14133087

[44] Rajfer J Ehrlich RM Goodwin WE Reduction clitoroplasty via ventral approach. J Urol (1982) 128:341. 7109104 10.1016/s0022-5347(17)52916-x

[45] de Jong TP Boemers TM. Neonatal management of female intersex by clitorovaginoplasty. J Urol (1995) 154:830. 10.1097/00005392-199508000-00135 7609190

[46] Schober JM Meyer-Bahlburg HF Ransley PG. Self-assessment of genital anatomy, sexual sensitivity and function in women: implications for genitoplasty. BJU Int (2004) 94:589. 10.1111/j.1464-410X.2004.05006.x 15329118

[47] Hutson J Voigt R Luthra M Kelly JH Fowler R Girth-reduction clitoroplasty – a new technique: experience with 37 cases. Pediatr Surg Int (1991) 6:336 10.1007/BF00178650

[48] O’Connell HE DeLancey JO. Clitoral anatomy in nulliparous, healthy, premenopausal volunteers using unenhanced magnetic resonance imaging. J Urol (2005) 173:2060. 10.1097/01.ju.0000158446.21396.c0 15879834 PMC1283096

[49] O’Connell HE Hutson JM Anderson CR Plenter RJ. Anatomical relationship between urethra and clitoris. J Urol (1998) 159:1892. 10.1016/S0022-5347(01)63188-4 9598482

[50] Rees MA O’Connell HE Plenter RJ Hutson JM. The suspensory ligament of the clitoris: connective tissue supports of the erectile tissues of the female urogenital region. Clin Anat (2000) 13:397. 10.1002/1098-2353(2000)13:6<397::AID-CA1>3.0.CO;2-2 11111889

[51] Lattimer JK Relocation and recession of the enlarged clitoris with preservation of the glans: an alternative to amputation. J Urol (1961) 86:113. 13759396 10.1016/S0022-5347(17)65118-8

[52] Randolph JG Hung W Reduction clitoroplasty in females with hypertrophied clitoris. J Pediatr Surg (1970) 5:224 10.1016/0022-3468(70)90279-4 5419072

[53] Fonkalsrud EW Kaplan S Lippe B. Experience with reduction clitoroplasty for clitoral hypertrophy. Ann Surg (1977) 186:221. 10.1097/00000658-197708000-00017 889369 PMC1396690

[54] Newman K Randolph J Parson S. Functional results in young women having clitoral reconstruction as infants. J Pediatr Surg (1992) 27:180. 10.1016/0022-3468(92)90308-T 1564615

[55] Pippi Salle JL Braga LP Macedo N Rosito N Bagli D. Corporeal sparing dismembered clitoroplasty: an alternative technique for feminizing genitoplasty. J Urol (2007) 178:1796. 10.1016/j.juro.2007.03.167 17707426

[56] Chia WY. Tissue-preserving feminizing clitoroplasty: a preliminary report. J Pediatr Urol (2007) 3:457. 10.1016/j.jpurol.2007.04.010 18947794

[57] Reifsnyder JE Stites J Bernabé KJ Galan D Felsen D Poppas DP Nerve sparing clitoroplasty is an option for adolescent and adult female patients with congenital adrenal hyperplasia and clitoral pain following prior clitoral recession or incomplete reduction. J Urol (2016) 195:1270 10.1016/j.juro.2015.12.053 26926549

[58] Alizai NK Thomas DF Lilford RJ Batchelor AG Johnson N. Feminizing genitoplasty for congenital adrenal hyperplasia: what happens at puberty? J Urol (1999) 161:1588. 10.1097/00005392-199905000-00071 10210421

[59] Passerini-Glazel G. A new 1-stage procedure for clitorovaginoplasty in severely masculinized female pseudohermaphrodites. J Urol (1989) 142:565. 2746778 10.1016/s0022-5347(17)38817-1

[60] Gearhart JP Burnett A Owen JH Measurement of pudendal evoked potentials during feminizing genitoplasty: technique and applications. J Urol (1995) 153:486 10.1097/00005392-199502000-00067 7815630

[61] Gastaud F Bouvattier C Duranteau L Brauner R Thibaud E Kutten F Impaired sexual and reproductive outcomes in women with classical forms of congenital adrenal hyperplasia. J Clin Endocrinol Metab (2007) 92:1391. 10.1210/jc.2006-1757 17284631

[62] Nordenskjöld A Holmdahl G Frisén L Falhammar H Filipsson H Thorén M Type of mutation and surgical procedure affect long-term quality of life for women with congenital adrenal hyperplasia. J Clin Endocrinol Metab (2008) 93:380. 10.1210/jc.2007-0556 18029470

[63] Jääskeläinen J Voutilainen R. Long-term outcome of classical 21-hydroxylase deficiency: diagnosis, complications and quality of life. Acta Paediatr (2000) 89:183. 10.1111/j.1651-2227.2000.tb01213.x 10709888

[64] Kuhnle U Bullinger M Schwarz HP. The quality of life in adult female patients with congenital adrenal hyperplasia: a comprehensive study of the impact of genital malformations and chronic disease on female patients life. Eur J Pediatr (1995) 154:708. 10.1007/BF02276713 8582420

[65] Berenbaum SA Korman Bryk K Duck SC Resnick SM. Psychological adjustment in children and adults with congenital adrenal hyperplasia. J Pediatr (2004) 144:741. 10.1016/S0022-3476(04)00237-9 15192620

[66] Wisniewski AB Migeon CJ Malouf MA Gearhart JP. Psychosexual outcome in women affected by congenital adrenal hyperplasia due to 21-hydroxylase deficiency. J Urol (2004) 171:2497. 10.1097/01.ju.0000125269.91938.f7 15126884

[67] Binet A Lardy H Geslin D Francois-Fiquet C Poli-Merol ML. Should we question early feminizing genitoplasty for patients with congenital adrenal hyperplasia and XX karyotype? J Pediatr Surg (2016) 51:465. 10.1016/j.jpedsurg.2015.10.004 26607969

[68] Cassia Amaral R Inacio M Brito VN Bachega TA Oliveira AA Jr Domenice S Quality of life in a large cohort of adult Brazilian patients with 46,XX and 46,XY disorders of sex development from a single tertiary centre. Clin Endocrinol (Oxf) (2015) 82:274. 10.1111/cen.12572 25074426

[69] Crawford JM Warne G Grover S Southwell BR Hutson JM. Results from a pediatric surgical centre justify early intervention in disorders of sex development. J Pediatr Surg (2009) 44:413. 10.1016/j.jpedsurg.2008.10.101 19231546

[70] Eckoldt-Wolke F. Timing of surgery for feminizing genitoplasty in patients suffering from congenital adrenal hyperplasia. Endocr Dev (2014) 27:203. 10.1159/000363664 25247657

[71] Mouriquand PD Gorduza DB Gay CL Meyer-Bahlburg HF Baker L Baskin LS Surgery in disorders of sex development (DSD) with a gender issue: if (why), when, and how? J Pediatr Urol (2016) 12:139 10.1016/j.jpurol.2016.05.040 27132944

